# A double-stranded RNA platform is required for the interaction between a host restriction factor and the NS1 protein of influenza A virus

**DOI:** 10.1093/nar/gkz1094

**Published:** 2019-11-22

**Authors:** Guifang Chen, Li-Chung Ma, Shanshan Wang, Ryan L Woltz, Emily M Grasso, Gaetano T Montelione, Robert M Krug

**Affiliations:** 1 Department of Molecular Biosciences, John Ring LaMontagne Center for Infectious Disease, Institute for Cellular and Molecular Biology, University of Texas at Austin, Austin, TX 78712, USA; 2 Center for Advanced Biotechnology and Medicine, Department of Molecular Biology and Biochemistry, Rutgers, The State University of New Jersey, Piscataway, NJ 08854, USA; 3 Department of Biochemistry and Molecular Biology, Robert Wood Johnson Medical School, Rutgers, The State University of New Jersey, Piscataway, NJ 08854, USA; 4 Center for Biotechnology and Interdisciplinary Studies, and Department of Chemistry and Chemical Biology, Rensselaer Polytechnic Institute, Troy, NY 12180, USA

## Abstract

Influenza A viruses cause widespread human respiratory disease. The viral multifunctional NS1 protein inhibits host antiviral responses. This inhibition results from the binding of specific cellular antiviral proteins at various positions on the NS1 protein. Remarkably, binding of several proteins also requires the two amino-acid residues in the NS1 N-terminal RNA-binding domain (RBD) that are required for binding double-stranded RNA (dsRNA). Here we focus on the host restriction factor DHX30 helicase that is countered by the NS1 protein, and establish why the dsRNA-binding activity of NS1 is required for its binding to DHX30. We show that the N-terminal 152 amino-acid residue segment of DHX30, denoted DHX30N, possesses all the antiviral activity of DHX30 and contains a dsRNA-binding domain, and that the NS1-DHX30 interaction *in vivo* requires the dsRNA-binding activity of both DHX30N and the NS1 RBD. We demonstrate why this is the case using bacteria-expressed proteins: the DHX30N-NS1 RBD interaction *in vitro* requires the presence of a dsRNA platform that binds both NS1 RBD and DHX30N. We propose that a similar dsRNA platform functions in interactions of the NS1 protein with other proteins that requires these same two amino-acid residues required for NS1 RBD dsRNA-binding activity.

## INTRODUCTION

Influenza A viruses cause an annual contagious respiratory human disease, and are responsible for periodic pandemics that result in high mortality (1). The influenza A virus genome is comprised of eight segments of negative sense viral RNA (2). The smallest segment encodes the NS1 protein, a small nonstructural protein that plays many crucial roles in virus infection, including inhibiting host antiviral responses, regulating other cellular and viral functions, and impacting virulence and virus-induced pathogenesis (3,4). The multiplicity of crucial NS1 functions highlights the importance of establishing the molecular mechanisms by which the NS1 protein carries out these functions.

One important function of the NS1 protein is the inhibition of host mRNA synthesis by binding a cellular 3′ end processing factor, the 30 kDa subunit of the cleavage and polyadenylation specificity factor (CPSF30) (5). Previously we purified CPSF30–NS1 complexes by sequential affinity selection of CPS30 and NS1 and identified the associated host proteins by mass spectrometry (6). Our results indicated that there are multiple NS1–CPSF30 complexes that differ with respect to the cellular protein(s) that are bound to the NS1 protein. One cellular protein associated with these complexes is the DDX21 RNA helicase, a host restriction factor that binds the PB1 viral polymerase subunit, thereby suppressing viral RNA synthesis and hence viral protein synthesis at early times after infection (6). DDX21-mediated antiviral activity is countered by the NS1 protein, which binds DDX21 and displaces PB1 from DDX21. These results prompted us to examine the potential antiviral activities of other cellular proteins in NS1–CPSF30 complexes. Here we focus on the cellular DHX30 helicase that is associated with NS1–CPSF30 complexes (6).

Many functions of the NS1 protein, specifically including its inhibition of host antiviral responses, result from the binding of specific cellular or viral proteins at various positions on the NS1 protein (3,4). Notably, the binding of several different proteins to the viral NS1 protein also require the same two amino-acid residues (Arg at position 38 and Lys at position 41) in the NS1 N-terminal RNA-binding domain (RBD) (residues 1-73) that are also required for binding double-stranded RNA (dsRNA) (6–12). The molecular basis for the requirement of these two NS1 amino-acid residues for interactions of the NS1 protein with other proteins has not been elucidated. Here, we uncover the underlying molecular mechanism for this requirement for the interaction between the NS1 protein and the cellular DHX30 RNA helicase. We show that the N-terminal 152 amino-acid residue segment of DHX30 (denoted as DHX30N) possesses all the antiviral activity of DHX30 and contains a dsRNA-binding domain. We also show that the dsRNA-binding activities of both DHX30 and the NS1 protein are required for the interaction of these two proteins, and that both dsRNA-binding activities are required because a dsRNA platform that binds both NS1 and DHX30 mediates the interaction between these two proteins. We propose that a similar dsRNA platform functions in the interactions of the NS1 protein with other proteins whose binding requires the dsRNA-binding amino acid-residues Arg38 and Lys 41 of the NS1 protein.

## MATERIALS AND METHODS

### Viruses and cells

HeLa, 293T and MDCK cells were grown in Dulbecco's modified Eagle's medium (DMEM) supplemented with 10% heat-inactivated fetal bovine serum. Wild-type (WT) Ud virus, and WSN virus were generated by plasmid-based reverse genetics as previously described (13). All eight genomic RNA segments of recombinant viruses were sequenced. Virus stocks were grown in 10-day-old fertilized eggs, and virus titers were determined by plaque assays using MDCK cells.

### siRNAs

The siRNAs against DHX30 was purchased from Thermo Scientific and resuspended in diethyl pyrocarbonate-treated water to a final storage concentration of 20 μM. The sequences of the two DHX30 siRNAs: (DHX30-1) GGAAGAGCUA-GAAGAAGGGACCAUA; (DHX30-2) CAAGGUGAUUCAGAUUGCAACGUCA. The control siRNAs were two different Stealth RNAi™ siRNA Negative Control siRNAs from Thermo Scientific.

### Plasmids and antibodies

The pcDNA3-HA-Flag-DHX30 and pcDNA3-HA-Flag plasmids were kindly provided by Guangxia Gao (14). The pcDNA3-HA-Flag-DHX30 plasmid encodes isoform 3 of DHX30. To generate a pcDNA3 plasmid expressing GST, the GST sequence in the pcNGST plasmid was amplified using appropriate primers, followed by XhoI and XbaI treatment. The DHX30 sequence in the pcDNA3-HA-Flag plasmid was amplified with appropriate primers, followed by BglII and XhoI treatment. These two DNA sequences were then ligated into BamHi and XbaI-treated pcDNA3 plasmid. To generate a pcDNA3 plasmid expressing the 150 amino acid-long N-terminal region of DHX30 (DHX30N), appropriate primers were used to amplify this region of DHX30 in the pcDNA3-HA-Flag-DHX30 plasmid. This DNA fragment was treated with SalI and BamHI, and then inserted into Sali and BglII-treated pcDNA3-HA-Flag plasmid. To generate the C region of DHX30 shown in Figure 3, this region was amplified using appropriate primers, followed by XhoI and BglII treatment and insertion into Sali and BglII-treated pcDNA3-HA-Flag plasmid. The pGEX-6P1 plasmids for bacterial expression of NS1 proteins were prepared as described previously (15). To generate pSRF plasmids for bacteria expression of His-tagged DHX30N, the N region of DHX30 in the pcDNA3-HA-Flag plasmid was amplified using appropriate primers, followed by BamHI and NotI treatment and insertion into the pSRF plasmid treated with these two restriction enzymes. Mutations were introduced using standard oligonucleotide mutagenesis methods. The antibodies (Abs) against NS1, PB1, PB2, PA and Flag were the same as described previously (6). Goat Ab against the major structural proteins of the Ud virus (HA (hemagglutinin), NP (nucleoprotein) and M1 (matrix protein) was kindly provided by Robert A. Lamb (designated Ud Ab) (16). DHX30 Ab was obtained from Abcam (ab85687).

### Immunoblots

Cell extracts were prepared as previously described (6). Briefly, cells were treated with a buffer containing 50 mM Tris–HCl (pH 7.5), 100 mM NaCl, 0.5% Nonidet P-40 and 1× protease inhibitor (Roche), followed by rotation of the extracts for 30 min at 4°C. The extracts were clarified by low-speed centrifugation at 4°C, and analyzed by immunoblots using the indicated Abs. Where indicated, the extracts were treated with 10 μg/ml of RNase A for 15 min at 37°C. In some experiments, the extracts were subjected to GST selection using glutathione magnet beads (Pierce), and the proteins eluted with glutathione were then immunoblotted; or FLAG-tagged proteins were selected using M2 Sepharose, and the proteins eluted with 3× FLAG were then immunoblotted.

### RNA binding assays

#### Gel shift assay

A 140-bp dsRNA was prepared by annealing the sense and anti-sense transcripts of a 140-nt long NS1 sequence inserted into the pGEM1 vector (10). This dsRNA (50 ng) was mixed with the amount of a wild-type or mutant DHX30N protein indicated in Figure 4 in a buffer containing 50 mM Tris–HCl (pH 7.5), 150 mM NaCl, 10% glycerol, 40U RNase in a total volume of 20 μl. The mixture was incubated on ice for 1 h and then subjected to electrophoresis on an agarose (1%) gel at 4 °C. The gel was stained with 1:10 000 SYBR-gold and scanned with typhoon scanner to detect RNA.

### Fluorescence polarization (FP) assay

A 16-nt single-strand (ss) RNA, FAM (fluorescein-labeled)- CCAUCCUCUACAGGCG (sense) was purchased from Dharmacon Inc. The FAM-labeled 16-bp dsRNA, the duplex of the sense and antisense FAM-labeled ssRNAs, were purchased from IDT. Fluorescence polarization (FP) was measured on a Tecan GENios-Pro plate reader with excitation at 485 nm and emission at 535 nm. FP values are reported in millipolarization units (mP). The reactions were carried out with 30 nM of 16-bp dsRNA or 16-nt sense ss-RNA with increasing concentrations of DHX30N in pH 6.0 nuclear magnetic resonance (NMR) Buffer (**below)**.

### Purification of bacteria-expressed DHX30N and NS1-RBD for biochemical and NMR studies

A DNA fragment encoding the NS1-RBD of Ud virus was cloned into the modified pSUMO vector (LifeSensors), as described previously (15), produced and purified as a N-terminal (6×His)-tagged SUMO fusion protein, and processed as outlined below to produce untagged NS1 RBD. The DNA fragment encoding DHX30N was generated by reverse transcriptase-polymerase chain reaction (RT-PCR) using human cDNA libraries, and cloned into bacterial expression vector NESG_ pET21 (15), with a C-terminal hexa-His tag. After sequence verification, each plasmid was transformed into *Escherichia coli* strain BL21(DE3) cells containing the rare tRNA expression plasmid pMCK (15). The same expression and purification protocols were used for SUMO-tagged NS1 RBD and DHX30N. Cells were first grown in LB media at 37°C to an absorbance of 0.6 units at 600 nm, and then isopropyl-β-D-thiogalactoside (IPTG) was added to a final concentration of 1 mM to induce the expression of the protein during 18 h of incubation at 17°C. The bacteria were harvested by centrifugation and resuspended in lysis buffer [50 mM Tris–HCl pH 7.5, 500 mM NaCl, 40 mM imidazole and 1 mM Tris-(2-carboxyethyl) phosphine], followed by mild sonication. After high-speed centrifugation, the supernatant was applied to a 5 ml HisTrap™ affinity column (GE Healthcare), which was washed with the lysis buffer, and the protein was eluted using elution buffer containing 50 mM Tris–HCl pH 7.5, 500 mM NaCl, 300 mM imidazole and 1 mM Tris-(2 carboxyethyl) phosphine. Further purification was performed using size exclusion chromatography on a HighLoad 26/60 Superdex S75 column (GE Healthcare) equilibrated in a buffer [50 mM Tris–HCl pH 8.0, 100 mM NaCl and 10 mM dithiothreitol (DTT)]. Additional steps were performed in order to obtain native NS1 RBD. The cleavage of the fusion protein of NS1-RBD was carried out by adding an aliquot in steps of 1:50 to 1:100 of yeast SUMO protease Ulp1 containing an N-terminal 6×His tag, and the sample was then incubated at 25°C for 18–24 h. The degree of cleavage was monitored by sodium dodecyl sulphate-polyacrylamide gel electrophoresis (SDS-PAGE). To remove nucleic acid and SUMO, the mixture was applied to a 5 ml HiTrap™ heparin column (GE Healthcare) and the NS1A RBD was separated from nucleic acid and SUMO by gradient elution with 0 to 1.0 M NaCl. Each preparation of purified NS1A RBD and DHX30N was >95% pure as determined by SDS-PAGE. Uniformly ^15^N enreiched NS1 RBD was produced using the same protocol, except that bacterial fermentation was done using MJ9 minimal medium (17) containing (^15^NH_4_)_2_SO_4_ as the sole nitrogen source. These samples were >95% pure, based on SDS-PAGE and > 98% ^15^N enriched based on MALDI-TOF mass spectrometry analysis.

### Co-expression of DHX30N and NS1-RBD in *E. coli*

A DNA fragment encoding NS1-RBD was cloned into the bacterial expression vector pET Duet RSF vector (Novagene), with a TEV protease cleavable maltose-binding protein (StrepII-MBP) fused at the N terminus. For DHX30N, the DNA fragment encoding residues 1–152 was generated by RT-PCR using human cDNA libraries, and cloned into a modified bacterial expression pET21c vector, NESG-pET21, with a C-terminal hexa-His tag with sequence LEHHHHHH to facilitate purification. The two plasmids were then transformed into *E. coli* strain BL21(DE3) cells containing the rare tRNA expression plasmid pMGK. The cells were first grown in LB media at 37°C to an absorbance of 0.6 at 600 nm, and then isopropyl-β-D-thiogalactoside was added to a final concentration of 1.0 mM and incubated for 18 h at 17°C to induce protein expression. The bacteria were harvested by centrifugation and resuspended in lysis buffer [50 mM Tris–HCl pH 7.5, 500 mM NaCl, 40 mM imidazole and 1 mM Tris-(2-carboxyethyl) phosphine)], followed by mild sonication. After high-speed centrifugation, the supernatant was applied to a 5 ml His-tag affinity column (GE Healthcare), which was eluted with a buffer containing 50 mM Tris–HCl pH 7.5, 500 mM NaCl, 300 mM imidazole and 1 mM Tris-(2-carboxyethylphosphine). The complex was then analyzed by size exclusion chromatography using a HighLoad 26/60 Superdex S75 column (GE Healthcare) in a buffer containing 50 mM Tris–HCl pH 8.0, 100 mM NaCl and 10 mM DTT. The fractions containing the complex of NS1 RBD/RNA/DHX30N were identified by absorbance at 280 nm and SDS-PAGE.

### DHX30N-NS1 RBD complex formation on Ni-NTA column

A 33-bp dsRNA with 2 nt overhangs on both 5′ and 3′ ends was prepared by annealing the following single-stranded RNAs purchased from Dharmacon Inc: CGAGCUCGCCCGGGGAUCCUCUAGAGUC-GACCUGC (sense) CUGCAGGUCGACUCUAGAGGAUCCCCGGGCGAGCU (antisense). A total of 66 μl of 20 μM His-tagged DHX30N with or without 40 μM dsRNA were incubated with 25 μl of Ni-NTA agarose resin (Qiagen) on each of two spin columns, in a loading buffer containing 20 mM sodium phosphate (pH 6.6), 100 mM NaCl, 20 mM β-mercaptoethanol and 1% glycerol at room temperature for 20 min. Both columns were then centrifuged at room temperature, and flow-through was collected. Next, 55 μl of 50 μM NS1-RBD (with no His tag) was loaded onto each of the two Ni-NTA spin columns to which DHX30N-H_6_ was bound and incubated in the same loading buffer for 20 min at room temperature. After the centrifugation and flow-through collection, each column was then washed twice with loading buffer. The bound species was eluted from each column using imidazole elution buffer described above. Fractions of the flow-throughs, washes and elutions were analyzed by SDS-PAGE.

### Modeling of DHX30N bound to dsRNA

Structural modeling of DHX30N was performed using the loop modeling software suite of Rosetta ver 3.3 (18,19). Models were generated using either DHX9 RNA binding domain 1 (residues 1–86; PDB_ID 3VYY) or DHX9 RNA binding domain 2 (residues 169–263; PDB_ID: 3VYX) as modeling templates. The SWISS-MODEL web server (20) was used to create multiple sequence alignments of DHX30N, DHX9 RNA binding domains 1 and 2, together with other homologous proteins. These alignments were then used to generate two homology models of DHX30N, using as templates either the DHX9 domain 1 (PDB ID: 3VYY) or domain 2 (PDB ID: 3VYX) coordinates. Gaps in the alignments between DHX9 domains and DHX30N were modeled using the *de novo* loop-modeling protocols of Rosetta (21). All gaps were modeled over a minimum of five amino-acid residues; in addition, we also included two amino-acid residues on either side of each gap when defining the loop region to model. The prediction confidence was increased by creating ∼10 000 decoys for each DHX30N model, and clustering the 1000 lowest energy models in this ensemble, as described elsewhere (19). These clusters were ranked based on the number of conformers in each cluster, the 10 highest ranked clusters were identified and the medoid conformer, i.e. the conformer with lowest backbone r.m.s.d. to each of the other models of each cluster (22), was selected as the representative conformer for that cluster. Each of these ten conformers generated from each template was then superimposed on the coordinates of corresponding DHX9 RNA binding domain bound to dsRNA. Because the dsRNA sequence bound to DHX9 RNA binding domain 2 is half as long as the dsRNA bound to DHX9 RNA binding domain 1, the dsRNA in the models generated from DHX9 RNA-binding domain 2 was replaced with the dsRNA coordinates of the DHX9 RNA-binding domain 2 complex. This provided two models of DHX30N: dsRNA with identical dsRNA lengths; each model consisting of an ensemble of 10 conformers. Molecular graphics and analyses were performed using the UCSF Chimera package (23).

### NMR Spectroscopy

NMR spectra of NS1-RBD, NS1-RBD/DHX30N, NS1RBD/dsRNA and NS1-RBD/DHX30N/dsRNA were carried out using samples protein concentrations of ca. 0.15 mM in pH 6.0 NMR Buffer containing 50 mM NH_4_OAc (pH 6.0), 200 mM NaCl, 50 mM Arg/Glu, 0.02% NaN_3_, 10% v/v ^2^H_2_O, except where noted otherwise. Spectra were acquired using a Bruker AVANCE 800 spectrometer equipped with a 5-mm TXI cryoprobe, thermostated at 298 K and referenced to internal DSS (2,2-dimethyl-2-silapentane-5-sulfonic acid). All NMR spectra were processed with *NMRPipe* software (24) and visualized using the program Sparky (25).

## RESULTS

### Cellular DHX30 RNA helicase inhibits influenza A virus replication by binding the viral NS1 protein

**Figure 1.**
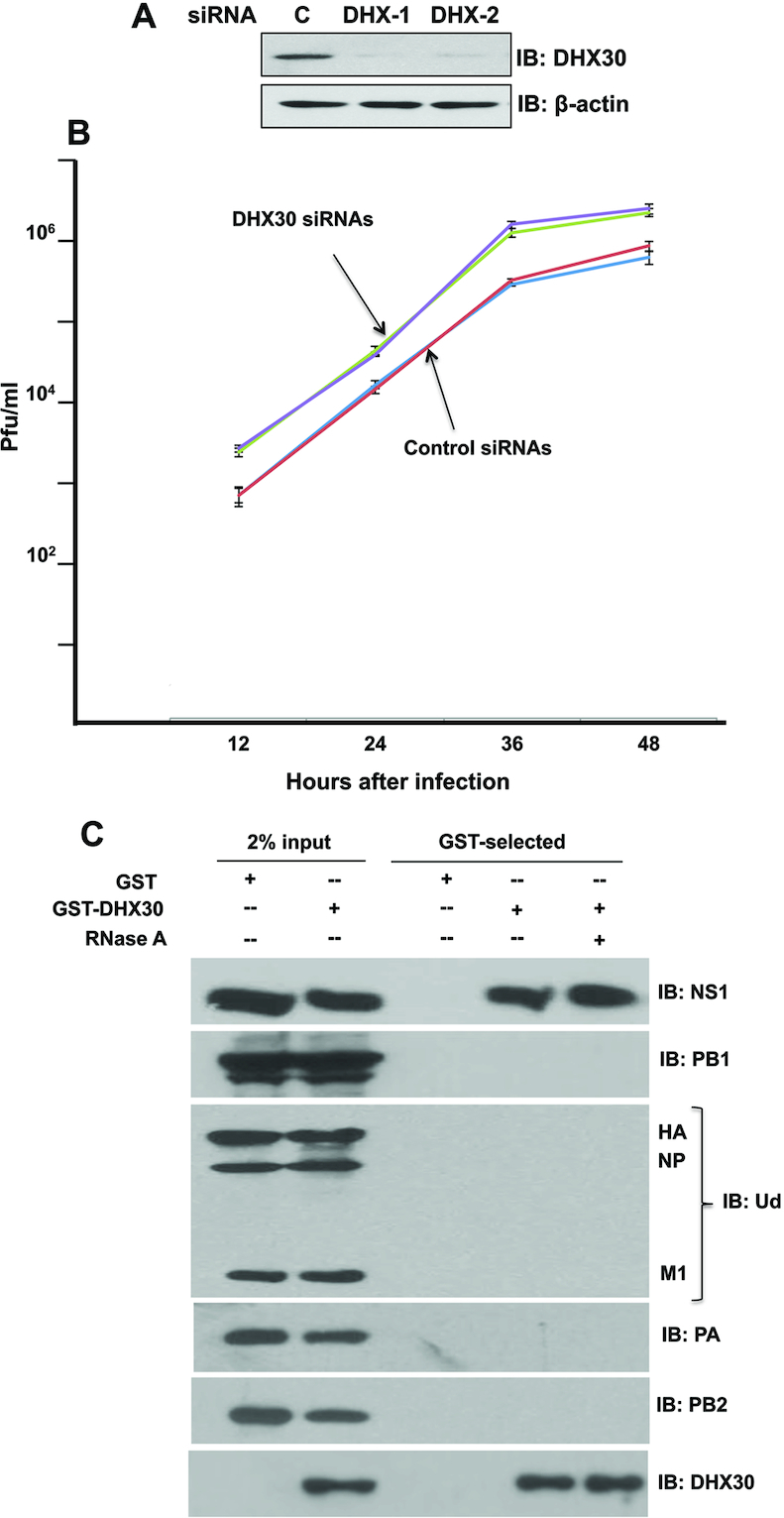
Cellular DHX30 RNA helicase inhibits influenza A virus replication by binding the viral NS1 protein. (**A**) Efficient knockdown of endogenous DHX30 in HeLa cells using DHX30-1 or DHX30-2 siRNA. Cell extracts were immunoblotted with the DHX30 antibody (Ab). (**B**) HeLa cells were transfected with the indicated DHX30 or control siRNAs for 36 h. The cells were then infected with 0.001 pfu/cell of Ud virus, followed by incubation at 37°C in serum-free DMEM medium supplemented with 1.0 μg/ml N-acetylated trypsin. Virus production at the indicated times after infection was determined by plaque assays in MDCK cells. These results and standard deviations (bars) are from three independent experiments. The virus titer at each time point in these three experiments was determined in triplicate plaque assays. Statistical significance between the viral growth curves in DHX30 siRNA-treated and control siRNA-treated cells was determined using a standard *t*-test, yielding a highly significant *P* value of 0.0043. (**C**) 293T cells were transfected for 24 h with a plasmid expressing GST-DHX30 or GST. The cells were then infected with 2 pfu/cell of Ud virus, followed by incubation at 37°C in serum-free DMEM medium. Cells collected at 12 h after infection were extracted as described in ‘Materials and Methods’ section. The extracts with or without treatment with RNase A were GST-selected as described in ‘Materials and Methods’ section. Aliquots of the eluates were analyzed by immunoblots probed with the indicated Abs to detect DHX30, and the following viral proteins: NS1, HA (hemagglutinin), NP (nucleoprotein), M1 (matrix protein) and the three subunits of the viral polymerase (PB1, PB2 and PA).

Previous results identified the cellular DHX30 RNA helicase in purified complexes containing the NS1 protein and the cellular CPSF30 3′ end processing factor (6). To determine whether endogenous DHX30 is indeed a host restriction factor that inhibits influenza A virus infection, human HeLa cells were transfected with two different DHX30 siRNAs, both of which efficiently knocked down DHX30 or with a control siRNA (Figure 1A). The three sets of cells, plus cells transfected with a different control siRNA, were infected with Ud virus at low multiplicity of infection (moi, 0.001 plaque forming units (pfu)/ml)) (Figure 1B). Replication of Ud virus was enhanced 5-8-fold in DHX30 knockdown cells compared to cells transfected with the control siRNAs, demonstrating that DHX30 inhibits influenza A virus replication.

To identify the viral proteins that bind DHX30, 293T cells were transfected for 24 h with a plasmid expressing GST-DHX30 and, as control, a plasmid expressing GST, followed by infection with 2 pfu/cell of Ud virus. At 12 h after infection, cell extracts with or without prior RNase A treatment were subjected to GST selection, followed by immunoblots to detect viral proteins. RNase A would be expected to digest single-stranded RNA regions under the conditions of this assay (26). As shown in Figure 1C, only the NS1 protein was specifically selected by GST-DHX30. RNase A treatment did not affect the interaction between NS1 and DHX30. This result indicates that DHX30 is associated with NS1–CPSF30 complexes at least in part via its interaction with the NS1 protein, and that DHX30 is not in the population of NS1-CPSF30 complexes that contain other viral proteins (6). The NS1 protein is undoubtedly associated with many different cellular proteins in various other NS1–CPSF30 complexes (6).

### The DHX30 binding site on the NS1 protein requires amino-acid residues R38 and K41 that are also required for RNA binding

**Figure 2.**
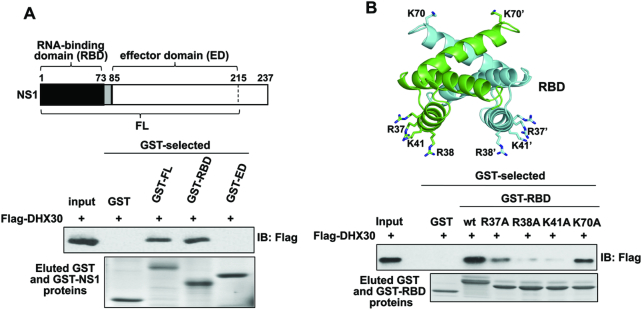
The DHX30 binding site on the NS1 protein is comprised of amino acid-residues R38 and K41 in the NS1 RBD. (**A**) (Top) Diagram of the regions of NS1 corresponding its RBD, ED and FL. The RBD and ED are linked by a short linker (shown in gray). The 215–237 disordered ‘C-terminal tail’ region was not included in the ED and FL plasmids because inclusion of this region greatly reduces the yield of soluble NS1 proteins. Bacteria-expressed GST or the indicated GST-tagged NS1 proteins were bound to glutathione magnetic beads, which were then incubated for 4 h with 293T cell extracts containing plasmid-expressed Flag-DHX30 that had been pretreated with RNase A. The proteins eluted from the beads using 20 mM glutathione were analyzed by immunoblots probed with Flag Ab and by Coomassie blue staining. (**B**) (top) Structure of the RBD showing the amino acid residues (R37/R37′, R38/R38′, K41/K41′, K70/K70′) that were replaced with alanines. The RBD structure was generated from Protein Data Bank (PBD ID: 1NS1) (43). (bottom) Bacteria extracts containing GST, GST-NS1 (1–73) or GST-NS1 (1–73) containing the indicated amino acid residue substitutions were bound to glutathione magnetic beads, which were then incubated with 293T cell extracts containing plasmid-expressed Flag-DHX30 that had been pretreated with RNase A. The eluted proteins were analyzed by immunoblots probed with Flag Ab and by Coomassie blue staining.

The NS1 protein contains two major domains: N-terminal RNA-binding domain (RBD, amino-acid residues 1–73); and effector domain (ED, amino-acid residues 85-C-terminus), which are connected by a short polypeptide linker (3,4) (Figure 2A). To determine which of these two NS1 domains bind DHX30, bacteria-expressed GST-RBD, GST-ED and GST-full-length (FL) NS1 proteins were affinity selected on glutathione magnetic beads. The beads containing these GST-tagged proteins, or the GST tag alone, were mixed with 293T cell extracts containing plasmid-expressed Flag-DHX30. The amount of DHX30 bound to the GST protein on the beads was assayed using an immunoblot probed with anti-Flag antibody. As shown in Figure 2A, DHX30 binds to FL NS1 and the RBD of the NS1 protein, but not to the ED of the NS1 protein.

To determine which amino-acid residues in the NS1 RBD are required for binding DHX30, we first focused on amino-acid residues that participate in dsRNA-binding (Arg37, Arg38, Lys41) (9,10) and, as a control, Lys70, a surface-exposed amino-acid residue in another region of the RBD (Figure 2B). Purified bacteria-expressed GST-RBDs in which each of these amino-acid residues was changed to alanine (A) were tested for binding Flag-DHX30. The RBD containing R37A bound substantially less DHX30 than wild-type (wt) RBD, and the RBDs containing either R38A or K41A bound only minimal amounts of DHX30, whereas the RBD containing the K70A substitution bound DHX30 almost as well as wt RBD. Consequently, the same amino-acid residues of the RBD, particularly R38 and K41, that are critical for dsRNA-binding are also critical for binding DHX30 in cell extracts.

### RNA-binding activity of the N-terminal domain of DHX30 is necessary and sufficient for binding the NS1 protein and inhibiting virus replication

**Figure 3.**
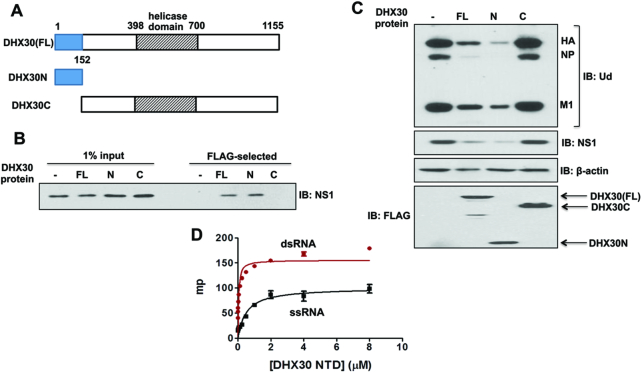
N-terminal 152 amino-acid residue-long domain of DHX30 (DHX30N), which has dsRNA-binding activity, is necessary and sufficient for binding the NS1 protein and inhibiting virus replication. (**A**) Diagram of FL DHX30, and N and C DHX30 protein regions. (**B**) 293T cells were transfected with a pcDNA3 plasmid expressing Flag-tagged FL, DHX30N, or an empty (−) pcDNA3 plasmid, followed by 12 h of infection with 2 pfu/cell of Ud virus. Cell extracts were treated with RNase A, and FLAG-tagged proteins were selected using M2 Sepharose, as described in ‘Materials and Methods’ section, followed by immunoblotting with NS1 Ab. (**C**) HeLa cells were transfected with an empty (-) pcDNA3 plasmid, or a pcDNA3 plasmid expressing FL, N or C DHX30 protein. Cells were infected for 6 h with 2 pfu/cell of Ud virus. Cell extracts were then immunoblotted with the indicated Abs. (**D**) A FAM (fluorescein-labeled)-16-nt ssRNA and FAM-16-bp dsRNA were incubated with increasing amounts of DHX30, and milli-polarization units (mp) were determined as described in ‘Materials and Methods’ section.

DHX30 has been reported in multiple isoforms. We used isoform 3 (UniProt ID Q7L2E3-3) that contains 1155 amino-acid residues beginning with N-terminal residues Met-Ala-Ala-Ser-Arg. Surprisingly a small (152 amino-acid residue-long) N-terminal fragment of DHX30, denoted as DHX30N (Figure 3A), was sufficient to bind the NS1 protein (Figure 3B). In addition, transfection of cells with a plasmid expressing DHX30N was as effective as transfection of a plasmid expressing full-length DHX30 in inhibiting influenza A virus replication (Figure 3C), demonstrating that the N fragment of DHX30 possesses all the antiviral activity of DHX30. The DHX30N domain is homologous to several known dsRNA-binding domains (pdb_id 2DB2) (27). We validated the dsRNA-binding activity of DHX30N using FP (Figure 3D). DHX30N binds dsRNA with a *K*_d_ of ∼40 nM, and also binds single-stranded RNA with a lower affinity (*K*_d_ of ∼500 nM).

**Figure 4.**
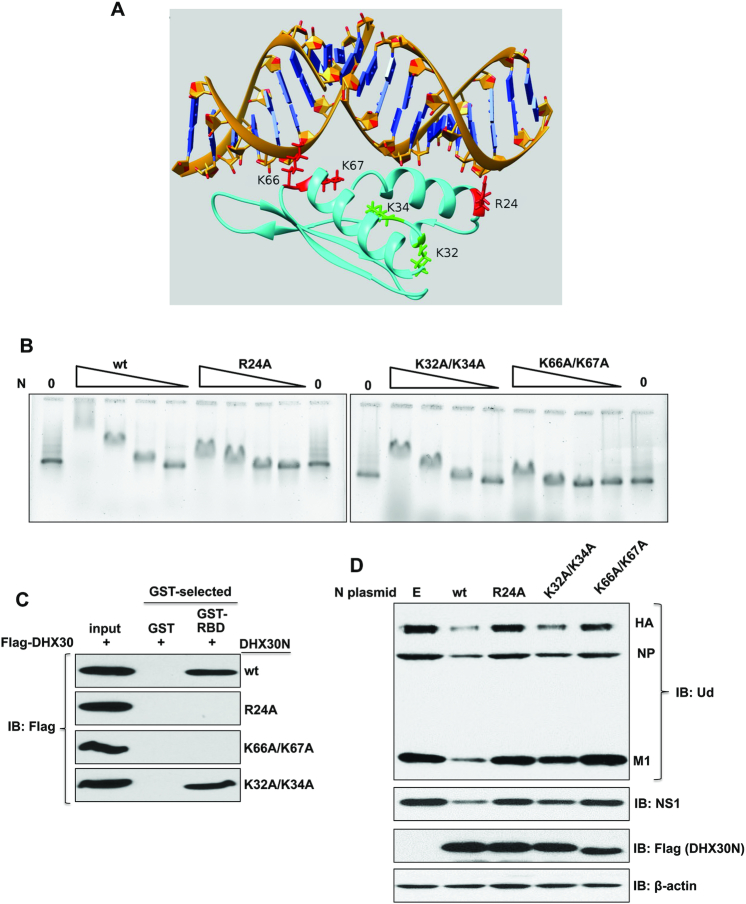
RNA-binding activity of DHX30N is necessary and sufficient for binding the NS1 protein and inhibiting virus replication. (**A**) Model of the DHX30N RNA-binding domain bound to dsRNA based on DHX9 RNA-binding domain 2 bound to dsRNA (3VYX). The structure of the complex shown corresponds to the single representative conformer of the top-ranked modeling cluster bound to dsRNA. The numbering of amino-acid residues starts with the Met-Ala-Ala-Ser-Arg N-terminal sequence of DHX30 isoform 3 (44). (**B**) Decreasing concentrations (36, 12, 4, 1.3 μM) of wt or the indicated mutant DHX30N protein were incubated with a 140-bp dsRNA for 1 h at 0°C, and the mixture was subjected to electrophoresis on a 1% agarose gel at 4°C. The gel was stained with SYBR-gold to identify RNA. (**C**) Bacteria-expressed GST or GST-tagged NS1-RBD was bound to glutathione magnetic beads, which were then incubated for 4 h with 293T cell extracts containing plasmid-expressed Flag-DHX30wt or Flag-DHX30 containing the indicated mutations in the N-terminal domain. These extracts had been treated with RNase A. Proteins eluted from the beads were analyzed by immunoblots probed with Flag Ab. (**D**) HeLa cells were transfected for 24 h with an empty (**E**) pcDNA3 plasmid, or a pcDNA3 plasmid expressing the indicated wt or mutant DHX30N protein. Cells were then infected for 6 h with 2 pfu/cell of Ud virus. Cell extracts were RNase A treated and then immunoblotted with the indicated Abs.

To identify the amino-acid residues that most likely interact with dsRNA, homology modeling was used to model the complex formed between DHX30N and dsRNA. Although the NMR structure of DHX30N has been determined (27), simple docking of this structure with dsRNA suggested that some small conformational changes occur upon dsRNA binding. Because DHX30N is homologous with the two dsRNA-binding domains of the DHX9 RNA helicase (> ∼30% sequence identity), and X-ray crystal structures of these two domains bound to dsRNA are available (PDB_ids 3VYX and 3VYY) (28), we used these X-ray structures to guide our modeling of DHX30N bound to dsRNA. In this modeling, as described in ‘Materials and Methods’ section, two models of the complex were generated; one based on DHX9 RNA-binding domain 1 bound to dsRNA (3VYY) and the other based on DHX9 RNA-binding domain 2 bound to dsRNA (3VYX). Figure 4A shows the model of the complex based on DHX9 RNA-binding domain 2 bound to dsRNA (3VYX).

This modeling study predicts, for both models, that basic sidechains of amino-acid residues Arg24 and Lys66 of the DHX30N RNA-binding domain interact with the dsRNA backbone. The sidechain of residue Lys67 is also close to the dsRNA backbone. In contrast, the Lys34 sidechain is not near the dsRNA backbone. Residue Lys32 is near the dsRNA backbone in the model generated using DHX9 RNA-binding domain 1, but not in the model using DHX9 RNA-binding domain 2 as the template shown in Figure 4A. Consequently, this modeling predicts that alanine substitution of Arg24, or of both Lys66 and Lys67 would strongly inhibit dsRNA binding, whereas alanine substitution of both Lys32 and Lys34 would have less effect on dsRNA binding. To test these predictions and to validate our model, we measured the binding of DHX30N proteins containing such alanine substitutions to a 140-base dsRNA using gel shift assays (Figure 4B). The R24A and K66A/K67A DHX30N mutant RNA-binding domains lost almost all dsRNA binding activities, as predicted by both models. Also, the K32A/K34A DHX30N mutant domain has less dsRNA-binding activity than the wt protein, as shown by the gel shift results at the two highest levels of the wt and K32A/K34A mutant proteins. These mutation results validate our models of the DHX30N/dsRNA complex.

Unlike the wt DHX30N protein, the R24A and K66A/K67A mutant DHX30N proteins that lack almost all dsRNA-binding activity did not bind the NS1 RBD in *in vivo* transfection experiments, as assayed in co-immunoprecipitation assays (Figure 4C), demonstrating that the dsRNA-binding activity of the DHX30N domain is required for DHX30N to bind the NS1 protein *in vivo*. In contrast, the K32A/K34A mutant DHX30N protein that has a considerably smaller reduction in dsRNA-binding activity still bound NS1RBD *in vivo*.

We next determined whether these mutant DHX30N proteins could inhibit virus replication by binding and sequestering the NS1 protein away from full-length DHX30 in virus-infected cells. We transfected an empty (E) plasmid, or plasmids expressing either wt or mutant DHX30N proteins into HeLa cells for 24 h, followed by infection for 6 h with 2 pfu/cell with Ud virus. Virus replication was assayed by measuring the levels of viral proteins (Figure 4D). Plasmid-driven overexpression of wt DHX30N protein strongly inhibited virus replication, whereas overexpression of the K66A/K67A mutant DHX30N protein lacking dsRNA binding activity had no detectable effect on virus replication, indicating that the dsRNA-binding activity of DHX30N is required for access of DHX30N to the NS1 protein in virus-infected cells. Interestingly, overexpression of the K32A/K34A mutant DHX30N protein that has a smaller reduction in dsRNA-binding activity only partially inhibited virus replication, indicating that only a DHX30N protein with full wt dsRNA-binding activity can optimally compete with wt full-length DHX30 protein for the NS1 protein in virus-infected cells. These results indicate that the dsRNA-binding activity of DHX30 is needed for its optimal binding to the NS1 protein that results in the inhibition of influenza virus replication.

### Interaction of bacteria-expressed NS1 RBD and DHX30N proteins *in vitro* requires the addition of dsRNA of sufficient length to bind both proteins

**Figure 5.**
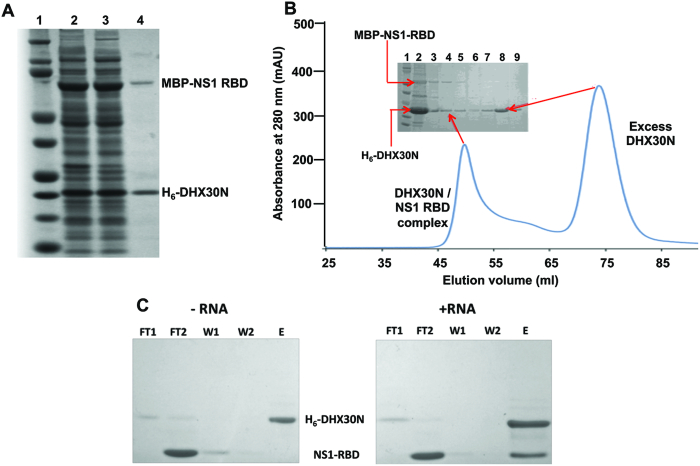
Formation of the DHX30N/NS1 RBD complex is dsRNA-dependent. (**A**) StrepII-MBP-NS1 RBD and His_6_-DHX30N (denoted as H_6_-DHX30N or more simply as DHX30N) were co-expressed in *Escherichia coli* Bl21(DE3) cells, and cell extracts were analyzed by SDS-PAGE. Lane 1. total cell extract. Lane 2 soluble cell extract. Lane 3. proteins purified from the soluble cell extract by Ni-NTA affinity selection. (**B**) Gel filtration chromatography of the proteins purified by Ni-NTA affinity selection. The inset shows the SDS-PAGE gel analysis of fractions from the gel filtration. Lane 1. molecular weight standards. Lane 2. Ni-NTA purified proteins applied to the column. Lanes 3 through 6. fractions from gel filtration with elution volumes of 45–55 ml. Lanes 7 through 9. fractions from gel filtration with elution volumes of 70–80 ml. (**C**). Purified His_6_-DHX30N was incubated with an excess of purified non-tagged NS1-RBD in the absence (left) or presence (right) of 33-bp dsRNA, and the mixture was applied to a Ni-NTA affinity column. SDS-PAGE analysis of proteins in: F1 and F2, two flow through fractions; E, imidazole eluate; W1 and W2, two wash fractions following imidazole elution.

We used bacteria-expressed proteins to determine why the interaction of the NS1 RBD with DHX30N requires the dsRNA-binding activity of both proteins. First, we determined whether NS1 RBD and DHX30N also formed a complex when co-expressed in bacteria. In these co-expression studies, NS1-RBD contained a N-terminal maltose-binding protein (MBP) with a Strep-II purification tag (designated as MBP-NS1 RBD), and DHX30N contained a C-terminal hexa-His (His_6_) tag. To determine whether a complex of these two proteins was formed when the proteins are co-expressed, the bacteria extract was subjected to Ni-NTA affinity chromatography to purify His_6_-DHX30N (Figure 5A). MBP-NS1 RBD was co-purified (lane 4), demonstrating that a complex containing both NS1-RBD and DHX30N was formed. Gel filtration chromatography of the Ni-NTA-selected His_6_-DHX30N further verified that this complex was formed: SDS-PAGE of the chromatography fractions identified this complex as well as the presence of an excess of DHX30N (Figure 5B). Fractions containing the DHX30N/NS1 RBD complex had A_260_/A_280_ ratios of 1.8 ± 0.2 absorbance units, indicating the presence of bound nucleic acid, while fractions containing excess DHX30N had A_260_/A_280_ ratios of 0.75 ± 0.05 absorbance units, values indicating a nucleic acid-free protein. These absorbance measurements are consistent with the presence of nucleic acid in the DHX30N/NS1 RBD complex.

To determine whether the interaction between NS1 RBD and DHX30N is indeed mediated by dsRNA, His_6_-DHX30N was loaded onto a Ni-NTA column in the presence or absence of a 33-bp dsRNA. Untagged NS1 RBD was then loaded onto each of the columns, which were washed to remove non-binding NS1 RBD. Bound proteins were eluted with imidazole and analyzed by SDS-PAGE (Figure 5C). Substantial amounts of NS1-RBD bound to the DHX30N:dsRNA complex (right), whereas little or no NS1 RBD was observed in the absence of dsRNA (left), demonstrating that DHX30N efficiently interacts with NS1 RBD in the presence of dsRNA, but not in its absence, and that both NS1 RBD and DHX30 interact with the same dsRNA molecule.

**Figure 6.**
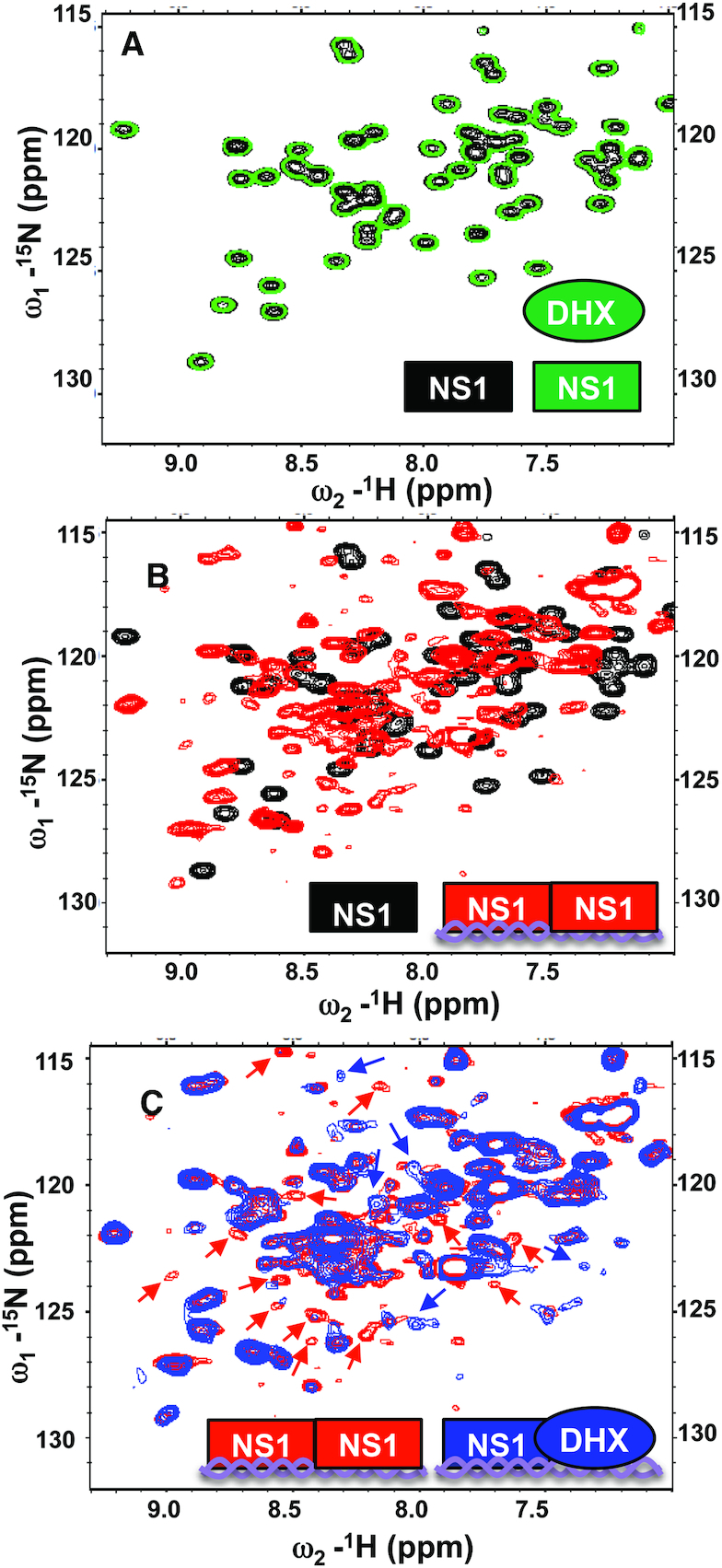
[^15^N-^1^H]-TROSY-HSQC fingerprint analysis of ^15^N-enriched non-tagged NS1 RBD (^15^N-NS1-RBD) demonstrates that the protein-protein interaction between DHX30N and NS1-RBD requires dsRNA. (**A**) Expanded region of the [^15^N-^1^H]-HSQC spectrum of ^15^N-NS1 RBD with (plotted in green) and without (plotted in black) unlabeled DHX30N. (**B**) Addition of unlabeled 33-nt dsRNA to ^15^N-NS1-RBD (spectrum plotted in black) results in extensive chemical shift perturbations due to complex formation (spectrum plotted in red). (**C**) Addition of unlabeled DHX30 to the ^15^N-NS1-RBD/dsRNA complex (spectrum plotted in red) results in additional chemical shift changes for ^15^N-NS1-RBD due new interactions of NS1-RBD, attributed to its binding to DHX30 in the complex. This same NMR study was carried out two times with independent samples. Resonances of the ^15^N-NS1-RBD/dsRNA complex which reproducibly disappear or broaden due to the addition of DHX30N are indicated with red arrows, and resonance which appear upon DHX30N binding are indicated with blue arrows. In each spectrum, the predominant species are illustrated schematically in the lower right in the color used to plot the corresponding spectrum; viz NS1-RBD (rectangle shape) DHX30N (oval shape), and dsRNA (double-stranded helix schematic). All NMR measurements were made using pH 6.0 NMR Buffer, defined in ‘Materials and Methods’ section.

To establish if there are protein-protein interactions between NS1 RBD and DHX30 in this complex, we used heteronuclear 2D NMR (nuclear magnetic resonance) spectroscopy of ^15^N-enriched NS1 RBD (^15^N-NS1 RBD) to probe for dsRNA-dependent interaction of NS1 RBD with DHX30N. ^1^H-detected ^15^N NMR allows observation of only the ^15^N-enriched NS1 RBD in these samples. As shown in Figure 6A, the ^15^N-heteronuclear transverse relaxation optimized spectroscopy (TROSY) single quantum coherence ([^15^N-^1^H]-TROSY-HSQC) fingerprint spectra of ^15^N-NS1B RBD were identical in the presence (green) or absence (black) of unenriched DHX30N, demonstrating that little or no interaction between these proteins occurred in the absence of dsRNA. In contrast, as shown in Figure 6B, the [^15^N-^1^H]-TROSY-HSQC spectra of ^15^N-NS1 RBD in the presence of an equimolar concentration of unlabeled 33-bp dsRNA (red) showed extensive amide ^15^N and ^1^H^N^ chemical shift changes compared to the spectra in the absence of dsRNA (black). These changes result from NS1-RBD binding to the dsRNA. When unenriched DHX30N was then added to the complex of ^15^N-NS1 RBD bound to dsRNA (spectrum plotted in red in Figure 6C) to form the multi-protein complex (spectrum plotted in blue in Figure 6C), several resonances of ^15^N-NS1 RBD broadened and/or shifted to new frequencies (red arrows), and several new peaks appeared (blue arrows). These new peaks do not correspond to resonances of unbound ^15^N-NS1 RBD shown in Figure 6A (black), demonstrating that these new resonances are not due to the displacement of ^15^N-NS1 RBD from dsRNA by unenriched DHX30N. Hence, we conclude that these NMR results show direct interactions between NS1 RBD and DHX30N domains in the NS1 RBD/dsRNA/DHX30 complex.

## DISCUSSION

Previous studies have demonstrated that the binding of several different proteins to the influenza A virus NS1 protein requires the same two amino-acid residues, Arg38 and Lys41, in the NS1 RBD that are also required for dsRNA binding (6–12). These observations suggested that binding of the NS1 RBD to dsRNA may be essential for the interaction of the NS1 protein with these proteins. Here we establish why NS1 dsRNA-binding is required for the interaction between NS1 and one host restriction factor, the DHX30 RNA helicase. First, we show that the dsRNA-binding activity of not only the NS1 protein but also the DHX30 helicase are required for their interaction *in vivo*. *In vitro* reconstitution experiments using bacteria-expressed proteins established that the protein-protein interaction of the DHX30N domain with NS1 RBD requires the presence of a 33-bp dsRNA that binds both proteins. The two proteins do not interact in the absence of this dsRNA. These results establish that a dsRNA platform that binds both NS1 and DHX30 is required for the interaction between these two proteins.

We propose that a similar RNA platform functions in the interactions of the NS1 protein with other proteins whose binding requires Arg38 and Lys41 of NS1. These other proteins include not only cellular proteins, for example, DDX21 RNA helicase (6) and TRIM25 E3 ligase (7,12), but also the viral nucleoprotein (NP) (8). TRIM25 binding to RNA has been shown to be required for many of its activities (29), suggesting that the interaction of TRIM25 with the NS1 protein requires that both proteins bind to dsRNA. To establish how dsRNA facilitates the interaction of the NS1 protein with these viral and cellular proteins, future structural studies are needed to reveal the detailed molecular interactions between the three components in a complex containing the NS1 protein, a specific targeted protein and dsRNA. Although an X-ray crystal structure of a complex formed *in vitro* between fragments of only the NS1 and TRIM25 proteins has been reported (30), this study does not exclude the enhancement by dsRNA of the formation of a functional complex between the corresponding full-length proteins, as is the case in virus-infected cells (7,12).

A dsRNA platform would be expected to increase the repertoire of proteins that interact with the NS1 protein in virus-infected cells because this platform would likely enable the NS1 protein to interact with proteins that lack sufficient affinity for effective NS1 protein binding in the absence of dsRNA. In addition, such a requirement for the dsRNA-binding activity of the NS1 protein would be one reason why amino-acid residues Arg38 and Lys41 of the NS1 protein that mediate dsRNA-binding are required for influenza virus replication (3–4,31–32). Other functions of the NS1 RNA-binding activity in virus-infected cells also contribute to the requirement of this activity for virus replication, including inhibition of the 2′-5′ oligo (A) synthetase/RNase L pathway (3–4,31–32).

While the NS1 protein binds to specific double-stranded RNA regions of several cellular RNAs (33,34), its binding to dsRNA *per se* is non-specific. It binds to any dsRNA sequence, no matter its sequence or source, e.g. influenza virus-infected mammalian cells, uninfected mammalian cells, bacteria (3,4,9,10,11; present study). The results reported here indicate that this is also likely the case for the N-terminal RNA-binding domain of DHX30. In influenza A virus-infected cells double-stranded viral RNAs generated during viral RNA synthesis would most likely provide the major dsRNA platforms for the interaction between the NS1 protein and DHX30. In our co-expression experiments, the RNA is provided by the bacterial expression host. In our *in vitro* reconstitution experiment we provided a short synthetic dsRNA of 33-bps as an RNA platform, thereby ensuring that the two proteins, DHX30 and NS1 RBD, are situated on the dsRNA adjacent to each other. Consequently, short double-stranded viral RNAs in infected cells would be suitable platforms for establishing protein-protein interactions between DHX30 and the NS1 protein. Longer double-stranded viral RNAs in infected cells should also be suitable platforms because the NS1 protein oligomerizes along dsRNAs (35–37) and hence should access DHX30 even if DHX30 does not oligomerize in the same fashion. We established that the DHX30 and NS1 proteins that are on the same dsRNA molecule also acquire protein–protein interactions, as determined by NMR spectroscopy analysis. More extensive structural studies will be carried out in the future to reveal the detailed molecular interactions between the three components (DHX30N, NS1 RBD and dsRNA) in this complex.

We found that treatment of human cell extracts with RNase A prior to binding assays did not eliminate the interaction of DHX30 with the NS1 protein. In fact, as shown in Figure 1, RNase A treatment did not reduce this interaction. These results are not surprising considering that RNase A preferentially cleaves single-stranded RNA regions under the conditions that we employed (26), so that double-stranded RNA regions of various sizes would remain largely undigested. Instead of treating human cell extracts with several different nucleases under various conditions in an attempt to achieve complete digestion of RNA, we opted to carry out *in vitro* reconstitution assays using bacteria-expressed, purified DHX30N and NS1 RBD proteins, with and without a well-defined synthetic dsRNA molecule, to establish that an RNA platform is required for this DHX30–NS1 interaction.

DHX30 enhances the viral mRNA degradation activity of the ZAP protein that is mediated by its N-terminal zinc-finger domain (14). ZAP-mediated viral mRNA degradation activity inhibits the replication of several viruses, including influenza A virus (38–40). The binding of DHX30 by the NS1 protein shown in the present study would be expected to partially inhibit the influenza viral mRNA degradation activity catalyzed by ZAP. This binding should not affect the other ZAP activity that is specific for influenza A virus, the degradation of the viral PB2 and PA polymerase subunits mediated by the ZAP C-terminal PARP domain (41). Consequently, only a modest increase in virus replication would be expected by the binding of DHX30 by the NS1 protein. The observed modest increase (∼5- to 8-fold) in virus replication resulting from DHX30 knockdown (Figure 1B) is consistent with this expectation.

Further studies are needed to determine whether an RNA platform that facilitates protein-protein interactions, as is the case for the DHX30–NS1 protein interaction described here, is a widespread mode of protein–protein interactions not only for the interactions of the NS1 protein with viral and cellular proteins but also for other protein–protein interactions. In fact, recent findings suggest that this mode of protein may be widespread: TRIM25 binding to RNA has been shown to be required for many of its activities (29) and the binding of the Riplet E3 ligase with RIG-I takes place on a dsRNA platform (42).

## References

[1] Wright P.F., Neumann G., Kawaoka Y. Knipe DM, Howley PM Myxoviruses. Fields Biology. 2012; 6 edn Philidelphia, PA Lippincott Williams & Wilkins 1186–1243.

[2] Krug R.M., Fodor E. Webster RG, Monto AS, Braciale TJ, Lamb RA The virus genome and its replication. Influenza Textbook. 2013; 2nd edn West Sussex Wiley-Blackwell 57–66.

[3] Ayllon J., Garcia-Sastre A. The NS1 protein: a multitasking virulence factor. Curr. Top. Microbiol. Immunol. 2015; 386:73–107. 25007846 10.1007/82_2014_400

[4] Krug R.M., Garcia-Sastre A. Webster RG, Monto AS, Braciale TJ, Lamb RA The NS1 protein: a master regulator of host and viral functions. Influenza Textbook. 2013; 2nd edn West Sussex Wiley-Blackwell 114–132.

[5] Nemeroff M.E, Barabino S.M, Li Y., Keller W, Krug R. M. Influenza virus NS1 protein interacts with the cellular 30 kDa subunit of CPSF and inhibits 3′ end formation of cellular pre-mRNAs. Mol. Cell. 1998; 1:991–1000. 9651582 10.1016/s1097-2765(00)80099-4

[6] Chen G., Liu C.H., Zhou L., Krug R.M. Cellular DDX21 RNA helicase inhibits influenza A virus replication but is counteracted by the viral NS1 protein. Cell Host Microbe. 2014; 15:484–493. 24721576 10.1016/j.chom.2014.03.002 PMC4039189

[7] Gack M.U., Albrecht R.A., Urano T., Inn K.S., Huang I.C., Carnero E., Farzan M., Inoue S., Jung J.U., Garcia-Sastre A. Influenza A virus NS1 targets the ubiquitin ligase TRIM25 to evade recognition by the host viral RNA sensor RIG-I. Cell Host Microbe. 2009; 5:439–449. 19454348 10.1016/j.chom.2009.04.006 PMC2737813

[8] Robb N.C., Chase G., Bier K., Vreede F.T., Shaw P.C., Naffakh N., Schwemmle M., Fodor E. The influenza A virus NS1 protein interacts with the nucleoprotein of viral ribonucleoprotein complexes. J. Virol. 2011; 85:5228–5231. 21411538 10.1128/JVI.02562-10 PMC3126214

[9] Cheng A., Wong S.M., Yuan Y.A. Structural basis for dsRNA recognition by NS1 protein of influenza A virus. Cell Res. 2009; 19:187–195. 18813227 10.1038/cr.2008.288

[10] Wang W., Riedel K., Lynch P., Chien C.Y., Montelione G.T., Krug R.M. RNA binding by the novel helical domain of the influenza virus NS1 protein requires its dimer structure and a small number of specific basic amino acids. RNA. 1999; 5:195–205. 10024172 10.1017/s1355838299981621 PMC1369752

[11] Chien C.Y., Xu Y., Xiao R., Aramini J.M., Sahasrabudhe P.V., Krug R.M., Montelione G.T. Biophysical characterization of the complex between double-stranded RNA and the N-terminal domain of the NS1 protein from influenza A virus: evidence for a novel RNA-binding mode. Biochemistry. 2004; 43:1950–1962. 14967035 10.1021/bi030176o

[12] Meyerson N.R., Zhou L., Guo Y.R., Zhao C., Tao Y.J., Krug R.M., Sawyer S.L. Nuclear TRIM25 specifically targets Influenza virus ribonucleoproteins to block the onset of RNA chain elongation. Cell Host Microbe. 2017; 22:627–638. 29107643 10.1016/j.chom.2017.10.003 PMC6309188

[13] Twu K.Y., Noah D.L., Rao P., Kuo R.L., Krug R.M. The CPSF30 binding site on the NS1A protein of influenza A virus is a potential antiviral target. J. Virol. 2006; 80:3957–3965. 16571812 10.1128/JVI.80.8.3957-3965.2006 PMC1440456

[14] Ye P., Liu S., Zhu Y., Chen G., Gao G. DEXH-Box protein DHX30 is required for optimal function of the zinc-finger antiviral protein. Protein Cell. 2010; 1:956–964. 21204022 10.1007/s13238-010-0117-8 PMC4875121

[15] Acton T.B., Xiao R., Anderson S., Aramini J., Buchwald W.A., Ciccosanti C., Conover K., Everett J., Hamilton K., Huang Y.J. et al. Preparation of protein samples for NMR structure, function, and small-molecule screening studies. Methods Enzymol. 2011; 493:21–60. 21371586 10.1016/B978-0-12-381274-2.00002-9 PMC4110644

[16] Chen B.J., Leser G.P., Morita E., Lamb R.A. Influenza virus hemagglutinin and neuraminidase, but not the matrix protein, are required for assembly and budding of plasmid-derived virus-like particles. J. Virol. 2007; 81:7111–7123. 17475660 10.1128/JVI.00361-07 PMC1933269

[17] Jansson M., Li Y.C., Jendeberg L., Anderson S., Montelione G.T., Nilsson B. High-level production of uniformly 15N- and 13C-enriched fusion proteins in Escherichia coli. J. Biomol. NMR. 1996; 7:131–141. 8616269 10.1007/BF00203823

[18] Raman S., Vernon R., Thompson J., Tyka M., Sadreyev R., Pei J., Kim D., Kellogg E., DiMaio F., Lange O. et al. Structure prediction for CASP8 with all-atom refinement using Rosetta. Proteins. 2009; 77(Suppl. 9):89–99. 19701941 10.1002/prot.22540 PMC3688471

[19] Bonneau R., Strauss C.E., Baker D. Improving the performance of Rosetta using multiple sequence alignment information and global measures of hydrophobic core formation. Proteins. 2001; 43:1–11. 11170209 10.1002/1097-0134(20010401)43:1<1::aid-prot1012>3.0.co;2-a

[20] Kiefer F., Arnold K., Kunzli M., Bordoli L., Schwede T. The SWISS-MODEL Repository and associated resources. Nucleic Acids Res. 2009; 37:D387–D392. 18931379 10.1093/nar/gkn750 PMC2686475

[21] Wang C., Bradley P., Baker D. Protein-protein docking with backbone flexibility. J. Mol. Biol. 2007; 373:503–519. 17825317 10.1016/j.jmb.2007.07.050

[22] Tejero R., Snyder D., Mao B., Aramini J.M., Montelione G.T. PDBStat: a universal restraint converter and restraint analysis software package for protein NMR. J. Biomol. NMR. 2013; 56:337–351. 23897031 10.1007/s10858-013-9753-7 PMC3932191

[23] Pettersen E.F., Goddard T.D., Huang C.C., Couch G.S., Greenblatt D.M., Meng E.C., Ferrin T.E. UCSF Chimera–a visualization system for exploratory research and analysis. J. Comput. Chem. 2004; 25:1605–1612. 15264254 10.1002/jcc.20084

[24] Delaglio F., Grzesiek S., Vuister G.W., Zhu G., Pfeifer J., Bax A. NMRPipe: a multidimensional spectral processing system based on UNIX pipes. J. Biomol. NMR. 1995; 6:277–293. 8520220 10.1007/BF00197809

[25] Lee W., Tonelli M., Markley J. L. NMRFAM-SPARKY: enhanced software for biomolecular NMR spectroscopy. Bioinformatics. 2015; 31:1325–1327. 25505092 10.1093/bioinformatics/btu830 PMC4393527

[26] Struhl K. Ausubel FM, Brent R, Kingston RE, Moore DD, Seidman JG, Smith JA, Struhl K Ribonucleases. Current Protocols in Molecular Biology. 2003; NY John Wiley & Sons 419.

[27] Abe C., Muto Y., Inoue M., Kigawa T., Terada T., Shirouzu M., Yokoyama S. RIKEN Structural Genomics/Proteomics Initiative 2006; doi:10.221/pdb2db2/pdb. 18409554

[28] Fu Q., Yuan Y.A. Structural insights into RISC assembly facilitated by dsRNA-binding domains of human RNA helicase A (DHX9). Nucleic Acids Res. 2013; 41:3457–3470. 23361462 10.1093/nar/gkt042 PMC3597700

[29] Sanchez J.G., Sparrer K.M.J., Chiang C., Reis R.A., Chiang J.J., Zurenski M.A., Wan Y., Gack M.U., Pornillos O. TRIM25 binds RNA to modulate cellular anti-viral defense. J. Mol. Biol. 2018; 430:5280–5293. 30342007 10.1016/j.jmb.2018.10.003 PMC6289755

[30] Koliopoulos M.G., Lethier M., van der Veen A.G., Haubrich K., Hennig J., Kowalinski E., Stevens R.V., Martin S.R., Reis E.S.C., Cusack S. et al. Molecular mechanism of influenza A NS1-mediated TRIM25 recognition and inhibition. Nat. Commun. 2018; 9:1820. 29739942 10.1038/s41467-018-04214-8 PMC5940772

[31] Donelan N.R., Basler C.F., Garcia-Sastre A. A recombinant influenza A virus expressing an RNA-binding-defective NS1 protein induces high levels of beta interferon and is attenuated in mice. J. Virol. 2003; 77:13257–13266. 14645582 10.1128/JVI.77.24.13257-13266.2003 PMC296096

[32] Min J.Y., Krug R.M. The primary function of RNA binding by the influenza A virus NS1 protein in infected cells: Inhibiting the 2′-5′ oligo (A) synthetase/RNase L pathway. Proc. Natl. Acad. Sci. U.S.A. 2006; 103:7100–7105. 16627618 10.1073/pnas.0602184103 PMC1459024

[33] Qiu Y., Nemeroff M., Krug R.M. The influenza virus NS1 protein binds to a specific region in human U6 snRNA and inhibits U6-U2 and U6-U4 snRNA interactions during splicing. RNA. 1995; 1:304–316. 7489502 PMC1369083

[34] Zhang L., Wang J., Munoz-Moreno R., Kim M., Sakthivel R., Mo W., Shao D., Anantharaman A., Garcia-Sastre A., Conrad N.K. et al. Influenza virus NS1 protein-RNA interactome reveals intron targeting. J. Virol. 2018; 92:e01634-18. 30258002 10.1128/JVI.01634-18 PMC6258958

[35] Yin C., Khan J.A., Swapna G.V., Ertekin A., Krug R.M., Tong L., Montelione G.T. Conserved surface features form the double-stranded RNA binding site of non-structural protein 1 (NS1) from influenza A and B viruses. J. Biol. Chem. 2007; 282:20584–20592. 17475623 10.1074/jbc.M611619200

[36] Bornholdt Z.A., Prasad B.V. X-ray structure of NS1 from a highly pathogenic H5N1 influenza virus. Nature. 2008; 456:985–988. 18987632 10.1038/nature07444 PMC2798118

[37] Aramini J.M., Ma L.C., Zhou L., Schauder C.M., Hamilton K., Amer B.R., Mack T.R., Lee H.W., Ciccosanti C.T., Zhao L. et al. Dimer interface of the effector domain of non-structural protein 1 from influenza A virus: an interface with multiple functions. J. Biol. Chem. 2011; 286:26050–26060. 21622573 10.1074/jbc.M111.248765 PMC3138300

[38] Gao G., Guo X., Goff S.P. Inhibition of retroviral RNA production by ZAP, a CCCH-type zinc finger protein. Science. 2002; 297:1703–1706. 12215647 10.1126/science.1074276

[39] Bick M.J., Carroll J.W., Gao G., Goff S.P., Rice C.M., MacDonald M.R. Expression of the zinc-finger antiviral protein inhibits alphavirus replication. J. Virol. 2003; 77:11555–11562. 14557641 10.1128/JVI.77.21.11555-11562.2003 PMC229374

[40] Tang Q., Wang X., Gao G. The short form of the zinc finger antiviral protein inhibits influenza a virus protein expression and is antagonized by the Virus-Encoded NS1. J. Virol. 2017; 91:e01909-16. 27807230 10.1128/JVI.01909-16 PMC5215320

[41] Liu C.H., Zhou L., Chen G., Krug R.M. Battle between influenza A virus and a newly identified antiviral activity of the PARP-containing ZAPL protein. Proc. Natl. Acad. Sci. U.S.A. 2015; 112:14048–14053. 26504237 10.1073/pnas.1509745112 PMC4653199

[42] Cadena C., Ahmad S., Xavier A., Willemsen J., Park S., Park J.W., Oh S.W., Fujita T., Hou F., Binder M. et al. Ubiquitin-Dependent and -Independent Roles of E3 Ligase RIPLET in Innate Immunity. Cell. 2019; 177:1187–1200. 31006531 10.1016/j.cell.2019.03.017 PMC6525047

[43] Chien C.Y., Tejero R., Huang Y., Zimmerman D.E., Rios C.B., Krug R.M., Montelione G.T. A novel RNA-binding motif in influenza A virus non-structural protein 1. Nat. Struct. Biol. 1997; 4:891–895. 9360601 10.1038/nsb1197-891

[44] Ota T., Suzuki Y., Nishikawa T., Otsuki T., Sugiyama T., Irie R., Wakamatsu A., Hayashi K., Sato H., Nagai K. et al. Complete sequencing and characterization of 21,243 full-length human cDNAs. Nat. Genet. 2004; 36:40–45. 14702039 10.1038/ng1285

